# Detailed survey of an *in vitro* intestinal epithelium model by single-cell transcriptomics

**DOI:** 10.1016/j.isci.2024.109383

**Published:** 2024-03-01

**Authors:** Ran Ran, Javier Muñoz Briones, Smrutiti Jena, Nicole L. Anderson, Matthew R. Olson, Leopold N. Green, Douglas K. Brubaker

**Affiliations:** 1 Center for Global Health and Diseases, Department of Pathology, Case Western Reserve University, Cleveland, OH, USA; 2 The Blood, Heart, Lung, and Immunology Research Center, Case Western Reserve University, University Hospitals of Cleveland, Cleveland, OH, USA; 3 Weldon School of Biomedical Engineering, Purdue University, West Lafayette, IN, USA; 4 Department of Biological Sciences, Purdue University, West Lafayette, IN, USA; 5 Purdue Interdisciplinary Life Science Program, West Lafayette, IN, USA

**Keywords:** Cell biology, Expression study, Systems biology, Transcriptomics

## Abstract

The co-culture of two adult human colorectal cancer cell lines, Caco-2 and HT29, on Transwell is commonly used as an *in vitro* gut mimic, yet the translatability of insights from such a system to adult human physiological contexts is not fully characterized. Here, we used single-cell RNA sequencing on the co-culture to obtain a detailed survey of cell type heterogeneity in the system and conducted a holistic comparison with human physiology. We identified the intestinal stem cell-, transit amplifying-, enterocyte-, goblet cell-, and enteroendocrine-like cells in the system. In general, the co-culture was fetal intestine-like, with less variety of gene expression compared to the adult human gut. Transporters for major types of nutrients were found in the majority of the enterocytes-like cells in the system. TLR 4 was not expressed in the sample, indicating that the co-culture model is incapable of mimicking the innate immune aspect of the human epithelium.

## Highlights

• Identified epithelial-like cell subtypes in Caco-2/HT29 co-culture
• Co-culture resembles fetal, not adult, gut
• Key nutrient transporters like FATP4/GLUT2 absent
• Co-culture lacks TLR4, incapable of innate immune response

## Graphical abstract

**Figure:**
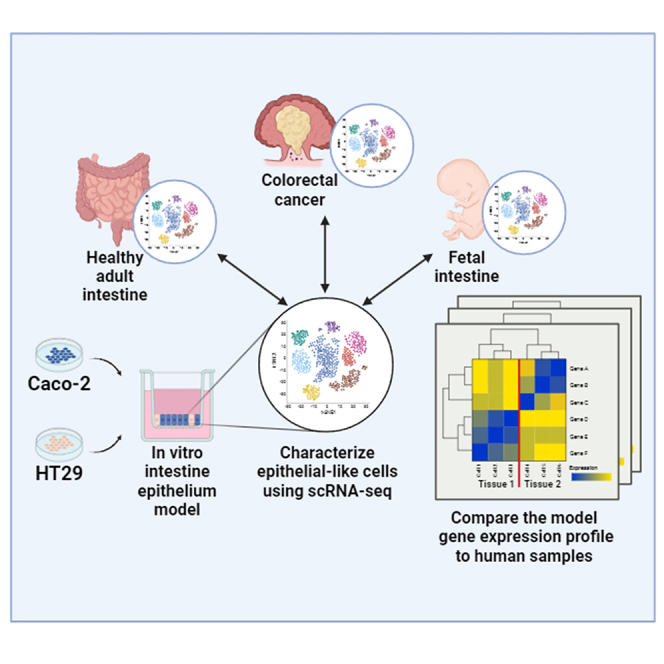

## Introduction

Inflammatory bowel diseases (IBD) are complex and debilitating diseases that affect millions of people worldwide.^1^ These disorders are characterized by an exaggerated immune response that leads to chronic inflammation in the gastrointestinal tract, resulting in a range of symptoms including abdominal pain, diarrhea, and rectal bleeding.^2^ The two most common forms of IBD are Crohn’s disease (CD), which can affect any part of the digestive tract and is characterized by transmural inflammation, and ulcerative colitis (UC), which is primarily confined to the colon and rectum and is characterized by continuous inflammation of the inner lining of the colon.^2^ The prevalence of IBD and colorectal cancer (CRC) in the United States has been increasing dramatically in recent years.^3^ According to recent estimates, approximately 3 million adults in the United States have been diagnosed with IBD, with an estimated annual healthcare cost of over $6 billion.^3^ This scenario is exacerbated by the higher risk of IBD patients relapsing within 1 year (28.7%) and 2 years (38.4%) after therapeutic de-escalation.^4^ Additionally, studies have shown that individuals with IBD have an increased risk of developing colorectal cancer, with the risk increasing with the duration and extent of the disease.^5^ Clearly, IBD is a significant health problem that requires urgent attention. Understanding the biology of IBD and the interactions between the immune system, the epithelium, and the microbiome is critical for the development of effective therapies for this disease.^2^

Caco-2 cells, derived from adult human colorectal adenocarcinoma, have been extensively employed as a remarkable proxy for the intricate gut epithelium.^6^ The salient features that render these cells an exemplary *in vitro* model include their ability to differentiate into enterocyte-like cells, recapitulating the intestinal absorptive characteristics.^6^ Furthermore, Caco-2 cells exhibit brush border enzymes, tight junctions, and a plethora of nutrient transporters, providing an accurate reflection of the physiological environment.^6^ Additionally, their robust and reproducible barrier function makes them indispensable for scrutinizing drug permeability and absorption studies.^7^ Lastly, the amenability of Caco-2 cells to various experimental manipulations bolsters their utility in exploring the multifaceted interactions between the gut epithelium and dietary components, pathogens, or pharmaceutical agents.^6^ On the other hand, HT29 cells, another adult human colon carcinoma cell line, have garnered significant attention as a valuable model for mimicking gut epithelial cells.^8^ These versatile cells exhibit the ability to differentiate into diverse intestinal cell types, including goblet and enteroendocrine cells, thus providing an authentic representation of the intestinal cellular milieu.^8^ Moreover, HT29 cells contribute to the understanding of mucus secretion dynamics, which play a critical role in maintaining gut barrier integrity and host defense.^9^

However, to sufficiently evaluate the translatability of insights from such a system to adult human physiological contexts, a detailed survey of cell type heterogeneity in the system and a holistic comparison with adult human physiology need to be conducted, not just by the presence of a few well-studied proteins.^10^ Single-cell RNA sequencing (scRNA-seq) represents a cutting-edge approach to meticulously assess the resemblance between *in vitro* gut mimics, such as Caco-2 and HT29 cells, and the authentic adult human intestinal epithelium.^11^ By offering a high-resolution transcriptomic landscape of individual cells, scRNA-seq facilitates the identification of distinct cell populations, thus enabling a comprehensive comparison of cellular composition and heterogeneity between these models and native tissue.^11^ Furthermore, this powerful technique uncovers the dynamics of gene expression and operative signaling pathways in both systems, shedding light on their functional congruence.^11^ Employing scRNA-seq in this context also aids in the discovery of potential limitations or disparities within *in vitro* models, fostering the refinement and optimization of these systems for enhanced physiological relevance.^12^ As a result, the subsequent improvements in these models pave the way for more accurate and insightful investigations of gut epithelial interactions with external factors, ultimately advancing our understanding of adult human gastrointestinal physiology and disease.^10^ In this study, we employed scRNA-seq to investigate the molecular signatures of the co-culture and assess its potential for producing results that can be generalized to the adult human gut. Transcriptional profiles of the co-cultured cells were acquired and compared to that of adult human epithelial cells,^13^ fetal gut epithelial cells,^14^ and human colorectal cancer cells,^15^ which were reported by previous studies.

## Result

### Determination of adult human cell type-like signatures represented in the germ-free co-culture model

We cultured Caco-2 (ATCC, passage no. 8) and HT29 (ATCC, passage no.13) separately at 1 × 105 cells/cm2 and seeded them together into the apical chamber of two Transwells (Corning Inc, Costar, USA) with a ratio of 9:1 on day 0. Cells were co-cultured and harvested on day 23. All samples were prepared for scRNA-seq as per the manufacturer’s instructions (10X genomics) (Figure S1A). Cell morphology agreed with the previous description of the Caco-2/HT29 monolayers (Figures S1A and S1B). The TEER values reported were within the range of values reported by Hoffmann et al.,^16^ indicating that the established Caco-2/HT29 co-culture system possesses properties comparable to those in other reported cases (Figure S1C).

**Figure 1:**
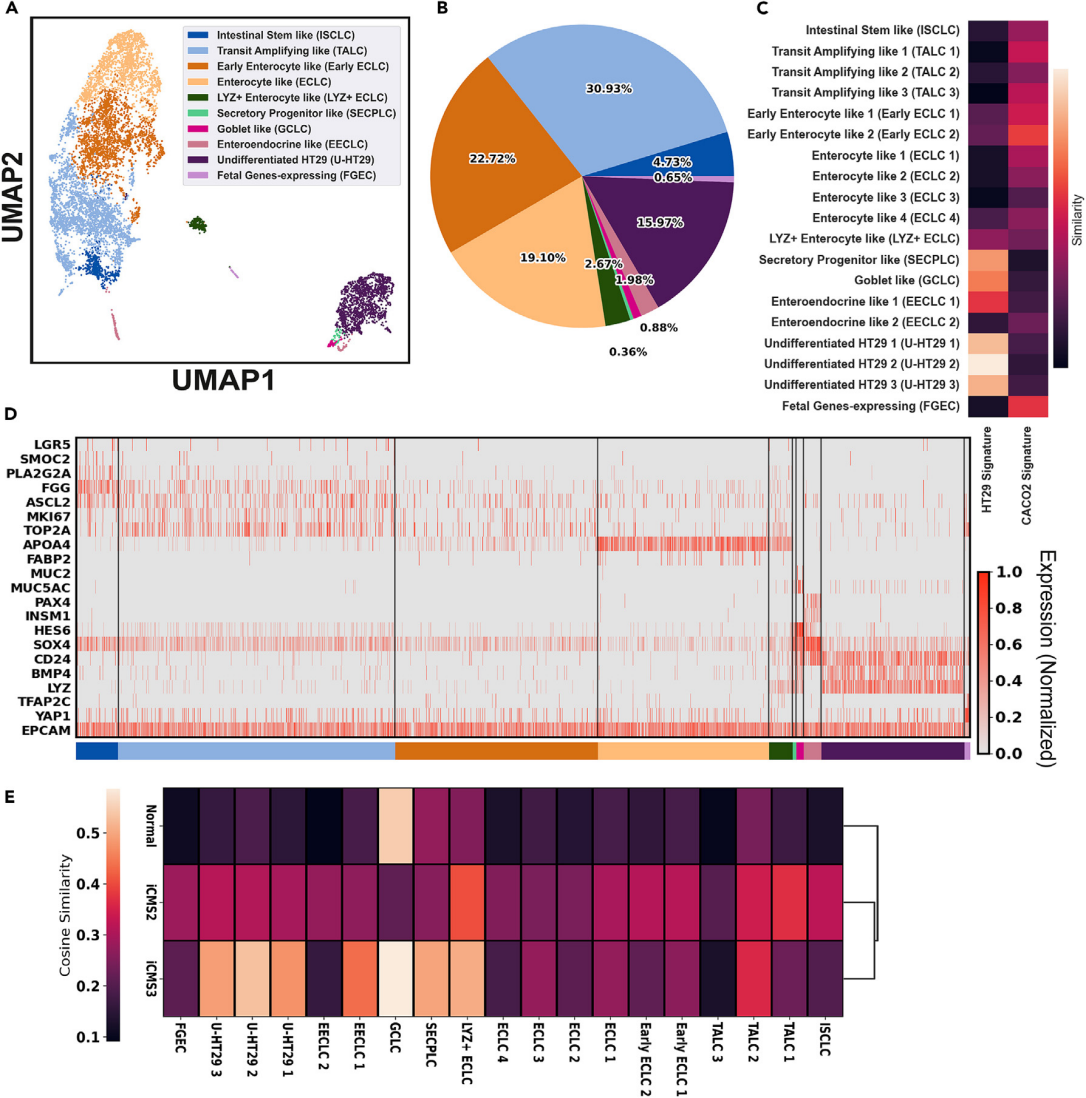
The Caco-2/HT29 co-culture model produces highly heterogeneous cell populations resembling intestinal epithelial lineage (A) Uniform manifold approximation and projection (UMAP) of cell populations in the co-culture model. ISCLC, intestinal stem-like cells; TALC, transit-amplifying-like cells; ECLC, enterocyte-like cells; SECPLC, secretory progenitor cell-like cells; GCLC, goblet cell-like cells; EECLC, enteroendocrine cell-like cells; U-HT29, undifferentiated HT29; FGEC, fetal genes expressing cells. (B) Composition of the co-cultured cells. (C) Conjectured Caco-2/HT29 origin of cell populations. Color bar: Jaccard similarity coefficient. (D) Heatmap of features in each cell population that have comparability with adult human intestinal epithelial lineages. (E) Heatmap showing the Cosine similarity of co-culture subsets with cell types isolated from the CRC patient.

**Figure 2:**
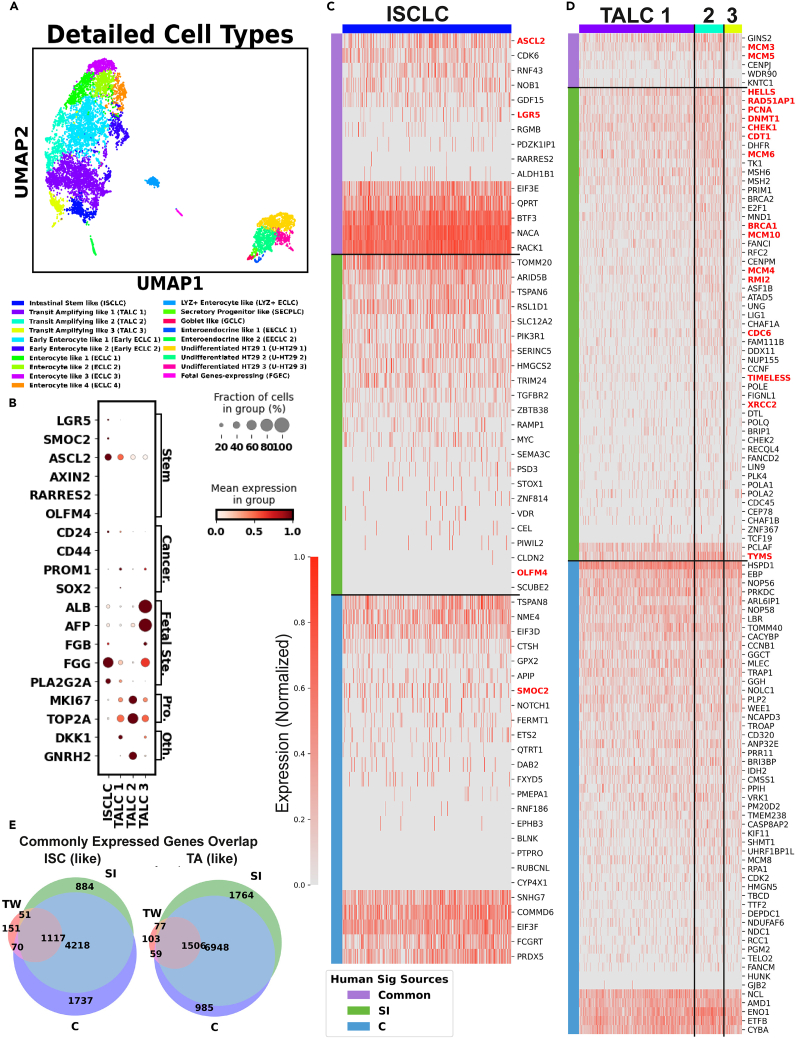
Characterization of ISCLC/TALC (A) UMAP of the ISCLC/TALC colored by detailed cell subtypes. (B) Dot plot showing the featured gene expression in each ISCLC/TALC subtype. (C and D) Heatmap showing the expression of adult human (C) ISC markers in ISCLC and (D) TA markers in TALC. (E) Venn diagram showing the overlap between commonly expressed genes (>30% of the cells) in ISCLC and adult human ISC (left) TALC and adult human TA (right).

Cell identities were annotated based on their expression of previously reported signatures (ISC: LGR5, ASCL2, OLFM4, SMOC2, OLFM4^17^; TA: MKI67, TOP2A^18^; EC: APOA4, ANPEP, FABP2^19^; GC: MUC2^20^; EEC: PAX4, INSM1, CHGA, CHGB^21^) in healthy adult human intestinal epithelial cells (Figure 1) and were further segmented into more detailed cell groups based on uniquely expressed genes (Figure 2A). Cell subsets with no similar known adult human analogy were annotated based on their cancer-related gene expression and differentiation state. Overall, the Caco-2/HT29 co-culture system was highly heterogeneous. Quantitatively, about 4.73% of the co-cultured cells were intestinal stem cell-like cells (ISCLC), 30.93% were transit amplifying-like cells (TALC), 44.49% were enterocyte-like cells (ECLC) where about half of them showed a conventional mature enterocyte feature, 0.36% were secretory progenitor-like cells, 1.98% were early enteroendocrine cell-like cells (EECLC), 0.88% were goblet cell-like cells (GCLC), and 15.97% were undifferentiated/differentiating HT29 cells (U-/D-HT29) (Figure 1G). There was also a small cluster (∼0.65% of the total cells) that exhibited fetal-like features, like the expression of TFAP2C (Table S1). The differentially expressed genes in each cluster were reported in Table S1. The conjectured origins of cell types, namely the monoculture Caco-2 or HT29, were determined by the overlap between cell type signatures and Caco-2/HT29 signatures (Table S2), identified from the bulk RNA-seq data of these two cell types provided by the Cancer Cell Line Encyclopedia (CCLE). Result showed that in this model, the enterocyte-like lineage was likely to derived from Caco-2 cells, and secretory-like lineage was more similar to the HT29 monoculture. Given their cancerous origin, we also calculated each subset’s cosine similarity with the normal, intrinsic consensus molecular subtype (iCMS) 2, and iCMS3 cells isolated from CRC patients reported in a previous study^15^ (Figure 1E). All co-culture subsets showed low similarity with the normal cells sampled from CRC except GCLC. In general, secretory-like cells presumably derived from HT29 had a more similar expression pattern with the iCMS3 CRC cells, and the ISCLC, TALC, ECLC in the model resemble the iCMS2 CRC more. We then asked how well the Caco-2/HT29 co-culture system can recapitulate the physiology of the adult human gut epithelium. We examined the expression profile of each major cell type in the model and asked if any of these properties disqualified the co-culture model for mimicking adult human gut epithelium.

### Characteristics of the stem and proliferative populations

Healthy adult human gut epithelium self-renews every 4–5 days. This quick turnover is initiated by the differentiation of ISCs located at the base of the crypt. Specifically, ISCs move upwards along the crypt-villi axis, leaving their stem niche and entering the region called transit-amplifying zone, where they rapidly proliferate and commit to either absorptive or secretory fate while transiting to the villus tip.^22^^,^^23^ In the co-culture, we identified 1 ISCLC subgroup and 3 TALC subgroups (Figure 2A). Noticeably, ISC and TA marker ASCL2, were universally expressed in all undifferentiated cells, which also agreed with the highly proliferative phenotype of cancer cells (Figure 2B).

ISCLC expressed fetal ISC marker FGG and classic intestinal stem markers LGR5 and SMOC2^17^ (Figure 2B). A portion of ISCLCs were also expressing ^22^ fetal/cancer marker alpha-fetoprotein (AFP)^24^^,^^25^ and its homologous albumin (ALB) (Figures 2B and 2C). Furthermore, ISCLC uniquely expressed PLA2G2A, a phospholipase A2 family member that is highly expressed in fetal ISC^26^ and adult adult human Paneth cells (PC).^27^ PLA2G2A is known to be a Wnt inhibitor and PC differentiation modulator,^28^ so its expression might indicate the Wnt activities and/or the identity of fetal small intestine ISCs or adult PCs. However, ISCLC did not express PC markers LYZ,^29^ DEFA4/5,^29^ or PRSS2^30^ (Figure S2C), so it was unlikely to be adult PC-like cells or the stem-like that have been reported to appear after inflammation in a previous study.^31^ Given the ISCLC also expressed^31^ CSC marker and embryonic development regulator CD24^32^^,^^33^ and BMP4^33^—although at a relatively low level—it is reasonable to comment on its similarity with adult human fetal ISCs. Since Wnt signaling maintains the self-renewal of ISCs,^34^ the active transcription of the Wnt inhibitor PLA2G2A in ISCLC might indicate it was at the beginning of differentiation and cue the loss of stemness in this population.

Compared to the ISCLC, TALCs had decreased ISC marker expressions and increased transcription of genes associated with proliferation such as MKI67 and TOP2A. The TALC 1 group highly expressed DKK1, a putative Wnt inhibitor,^35^ and YAP’s downstream ANKRD1.^36^ The potential Wnt inhibiting effect of DKK1 might signal the loss of stemness, thus agreed with the assigned TALC identity. Indeed, the activities of Wnt inhibitors in the stem/proliferative populations invite further investigation in the role of Wnt signaling pathway in the co-culture maintenance. We found the expression of genes that were engaged in the Wnt signaling, such as frizzled class receptor FZD 2/3/4/5/7^37^^,^^38^ as well as the transcription of several Wnt downstream genes like ASCL2, MYC,^38^ ID1,^39^ HES1,^40^ SOX9,^41^ etc. (Figure S2C; Table 1), which means the co-cultured cells were universally eligible of receiving Wnt signals and proceeding the downstream transcription. Despite the broad expression of Wnt-associated genes, Wnt ligands themselves were not highly expressed (Figure S2C). Such observation is reasonable, considering Paneth and mesenchymal cells are the main producers of Wnts in healthy adult human gut,^42^ and the co-culture model was sourced from the colon, which is considered Paneth-free, and did not have any stromal cells equivalent. Still, it raised the question how the Wnt pathway could be activated with such a limited Wnt secretion without the addition of exogenous Wnt (no Wnt protein in the culture medium). It is possible that the^43^ APC/beta-catenin mutation, which is well-characterized in both Caco-2 and HT29 cell lines,^43^^,^^44^ and/or the crosstalk between Wnt and other signaling pathways, such as TGF beta signaling,^45^ result in the activation of Wnt downstream.

TALC 2 exhibited high levels of MKI67 and TOP2A, but uniquely expressed gonadotropin-releasing hormone 2 (GNRH2), a hormone with unclear function. Unlike GNRH1, GNRH2 is commonly expressed in a variety of adult human peripheral tissues but is seldom found in brain regions associated with gonadotropin secretion,^46^ suggesting it might not function as a hormone in the same way GNRH1 does. Furthermore, it was observed that GNRH2 had an anti-proliferative effect on prostate, ovarian, breast, and endometrial cancer cells.^47^

The expression of AFP and ALB in TALC 3 was indicative of the resemblance of its features to the TA cells found in the fetal small intestine.^24^^,^^25^ This finding further supports the notion that the epithelium-like tissue derived from Caco-2/HT29 exhibits characteristics akin to the fetal intestine. Nevertheless, such finding was anticipated given the reported embryonic genes re-expressed or upregulated in colorectal adenocarcinoma.^48^^,^^49^

We examined the expression of previously reported adult human ISC/TA signatures in the model.^13^^,^^17^ Although LGR5 and SMOC2 were expressed, there was no active transcription of adult human adult ISC marker genes OLFM4, RARRES2, or CD44 (Figures 2B and 2C). Interestingly, the lack of OLFM4 is a feature of the immature fetal intestine, strengthening the fetal-like features of the co-culture^50^ (Figure S3A). However, we did not find any BEX5+ uniform progenitors^26^ that are reported to occur during the first trimester (Figure S2C), suggesting the features were not early fetal-like.

Overall, some of the adult human ISC differentially expressed genes (DEGs) identified in the previous study were not expressed in the ISCLC, and conversely, only a few of the ISCLC DEGs were expressed in adult human ISC (Figures 2C and S3A). A total of 1117 genes were expressed in over 30% of the ISCLC, adult human small intestine ISC, and adult human colon ISC, whereas 151 genes expressed in ISCLC were not commonly expressed in any of the adult human ISC (Figure 2E). Interestingly, more co-culture DEGs are expressed in fetal LGR5^+^ Stem cells (Figure S3A). By comparison, TALCs in the co-culture model shared more similarity with the adult human TA cells. Proliferative genes such as mini chromosome maintenance genes (MCMs) and cell division cycle (CDCs), which were differentially expressed in adult human TAs, were also prevalent in TALCs (Figure 2D). 1506 out of 1745 commonly expressed genes found in TALCs were also expressed in all adult human TAs regardless of their location in the gut (Figure 2E). It is also evident that most genes that were upregulated in TALC was also expressed in adult human TA (Figure S3D).

### Enterocyte-like cells and their absorption preference

**Figure 3:**
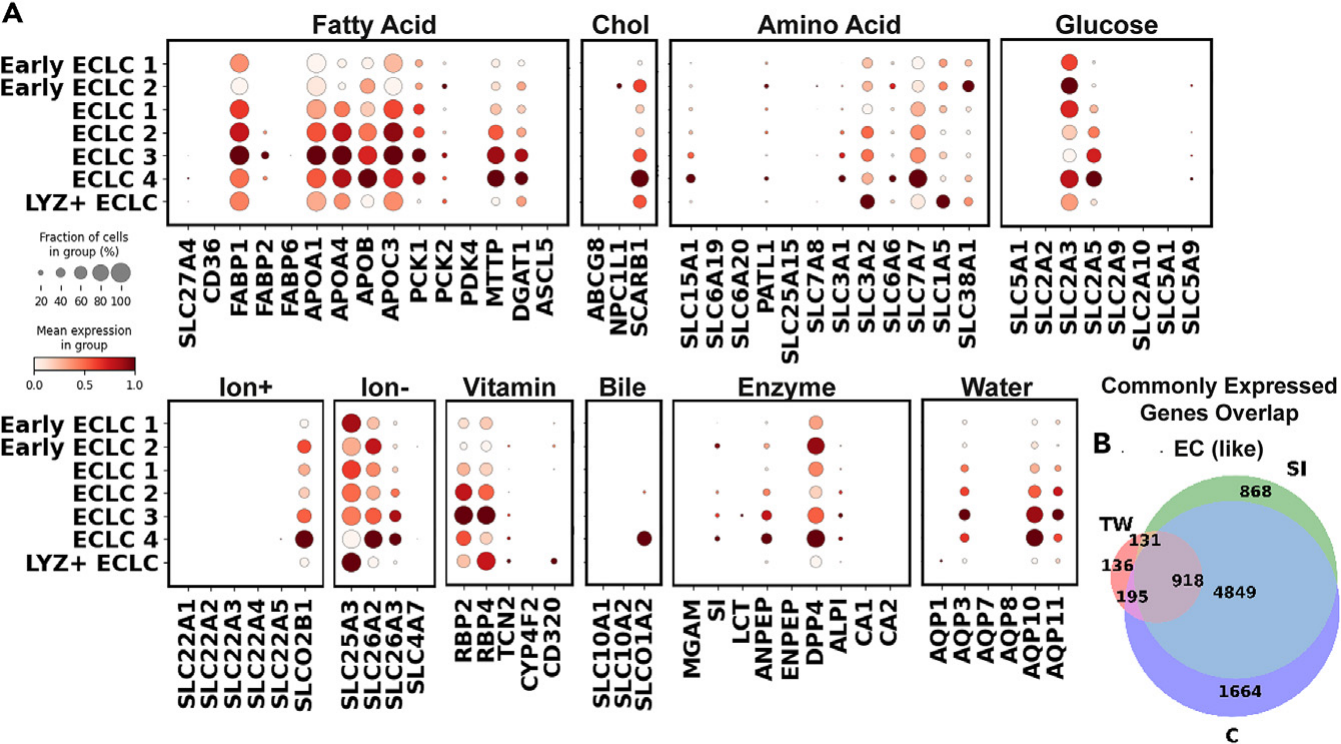
Characterization of ECLC (A) Dot plot showing the expression of key transporters in each ELCL subtype. Sig, signatures; Chol, cholesterol. (B) Venn diagram showing the overlap between commonly expressed genes (>30% of the cells) in ECLC and adult human enterocytes/colonocytes.

Enterocytes express a wide array of transporters, which play crucial roles in the absorption and secretion of nutrients, ions, and drugs, thereby maintaining the integrity of the intestinal barrier.^51^ Caco-2/HT29 system is a popular high-throughput model to study the drug intestinal absorption—especially drugs that are absorbed passively—and a variety of transporters have also been identified on the surface of the Caco-2 cells.^52^ However, the comprehensive profiling of transporters on Caco-2/HT29 remains incomplete due to the limited characterization methods available for the co-culture system during its establishment. Here, we identified 7 ECLC populations that resembles adult human enterocytes (Figure 3A). Their ECLC identity was defined by the expression of major mature enterocyte transporters APOA4, ANPEP, and FABP2.^13^^,^^49^^,^^51^ The degree of their differentiation was annotated based on the expression of proliferative markers in these cells. After the basic annotation, we queried the expression of 9 categories of functional proteins that are present and responsible for digestion and absorption in healthy adult human and fetal enterocytes (Figure S3C).

In our study, similar to the predominant genes in adult human ISC/TALC, the co-cultured cells showed limited transcriptional activity, particularly among ECLCs which displayed a narrower range of expressed fatty acid, glucose, amino acid transporters and enzymes compared to adult human and fetal enterocytes (Figures 3A and S3C). The co-cultured cells primarily expressed fatty acid transporters (APOA1/4, APOC3), a digestive enzyme (ANPEP, DPP4), components of vitamin retinol/retinaldehyde metabolism (RBP2/4), and water channels (AQP3/10/11) (Figure 3A). Significant transcription was also observed for the drug transporter ABCC2, bile acid transporter ABCC3, and cation transporter SLCO2B1 (OATP-B). Interestingly, these cells also transcribed SLC2A3 (GLUT3), a glucose transporter typically found in the brain, placenta, and CRC tissue.^53^^,^^54^ However, our co-culture system lacked several key transporters, including those for long-chain fatty acid absorption (SLC27A4,^55^ CD36^56^), cholesterol absorption (ABCG5/G8, NPC1L1),^57^ glucose absorption (SLC5A1, SGLT1, SLC2A2, GLUT2),^58^ drug intake (ABCB1), enzyme digestion (lactase), bile acid absorption (SLCO1A2, SLC10A2),^59^ water transportation (AQP1, 7, 8),^60^ and cation transportation (SLC22A1-5, SCNN1B).^61^ Taken together, we postulate that the metabolism of this group had been reprogrammed to involve certain cancer-related glycolysis burst events,^62^ which skews the co-culture model toward cancer metabolism. Consequently, the efficacy of certain drugs, especially those targeting the absent receptors, like long-chain fatty acid drugs (omega-3 fatty acids), lipid-lowering (Gemfibrozil/Bezafibrate), cholesterol-lowering (statins/ezetimibe), and glucose-lowering (sulfonylureas), might be inaccurately assessed in drug screenings and tests.

While the model exhibited more small intestine (SI) characteristics (i.e., active SI-specific genes with most colon-specific genes missing), all absorptive populations expressed high levels of colon-specific ion transporter SLC26A2, and vitamin transporter RBP4 were expressed in all absorptive populations, indicating the model also possesses colon-like features given both Caco-2 and HT29 were CRC-derived. 918 commonly expressed genes found in ECLC were also expressed in both the enterocytes and colonocytes (Figure 3B).

### Undifferentiated HT29, goblet cell-like cells, and immature enteroendocrine cell-like cells were present in the model

**Figure 4:**
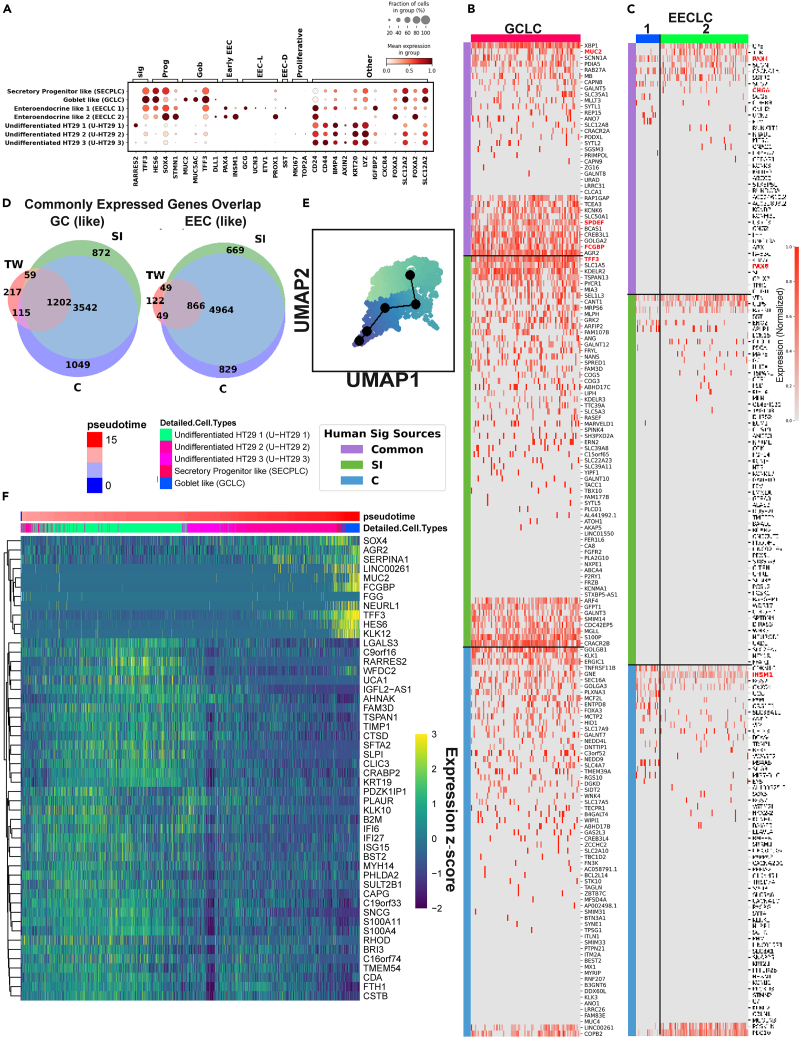
Characterization of secretory cell-like cells (A) Dot plot showing the featured gene expression in each HT29/secretory-like cells subtype. (B and C) Heatmap showing the expression of adult human (B) goblet markers in GCLC and (C) EEC markers in EECLC. (D) Venn diagram showing the overlap between commonly expressed genes (>30% of the cells) in GCLC and adult human SI and colon goblet cells (left) and EECLC and adult human SI and colon EEC (right). (E) UMAP of the HT29/secretory-like cells colored by pseudotime, light = early, dark = late. (F) Heatmap of gene expression changed along with the pseudotime. Expressions are standardized for each gene.

Undifferentiated HT29 cells, which are characterized by their rapid proliferation and ability to form a monolayer in culture without showing functional differentiation typical of mature enterocytes, such as the formation of microvilli and the expression of brush border enzymes. We found undifferentiated HT29 cells, secretory progenitor-like cells, goblet-like cells, and 2 groups of immature enteroendocrine-like cells present in the co-culture system (Figure 4A). Previous reports have shown that the majority (∼95%) of HT29 monoculture is in an undifferentiated state, and they were defined such that they do not express definitive sets of markers of known adult human epithelial cell types.^63^^,^^64^ Admittedly, the profile of undifferentiated HT29, if any exists in the co-culture, is likely to differ substantially from that of the monoculture due to communication with Caco-2 cell and Transwell geometry. Therefore, we assigned the identities to U-HT29 because of their lack of epithelial markers and share the most similarity with the profile of monoculture HT29 (Figures 1C–1E).

Most HT29 and secretory-like cells express CD24, CD44, AXIN2, and BMP4. CD24 and CD44, like discussed previously, are cell surface markers that are often used to identify cancer stem cells, which have the ability to self-renew and differentiate into various types of cancer cells. BMP4 is a bone morphogenetic protein that is reported to restrict the stemness of LGR5+ ISCs^65^ and enhance the differentiation.^66^ Such a BMP4 expression evokes similarity to Paneth or mesenchymal stromal cells because the currently accepted gut epithelial cells turnover model proposes that a BMP4 gradient increasing from the crypt to the villus tip guides the differentiation of ISCs, and a high level of BMP4 is found in the lamina propria of the villus tip, co-localizing with LGR5.^67^ However, the possibility of this population being stromal cells was undermined by the high expression of epithelial cell adhesion molecule (EPCAM). Interestingly, this population also highly expressed lysozyme (LYZ) and end-of-differentiation marker keratin 20 (KRT20).^68^ Lysozyme (LYZ), a well-studied antimicrobial agent, is transcribed in BEST4+ enterocytes, PCs, tuft cells, and follicle-associated epithelial cells, which seemingly supports the hypothesis that those cells being Paneth-like cells. Earlier studies also claimed HT29’s capability of differentiating into PCs based on its expression of LYZ.^69^ However, the lack of other Paneth markers DEFA4/5 or ITLN2 (Figure S2C) challenged the idea of this population being full-fledged PC-like cells.^7577^ Therefore, there is no functional mimic of PC or stromal cells in the Caco-2/HT29 co-culture.

SECPLC featured a high expression of fetal secretory marker HES6,^25^ secretory differentiation modulator SOX4,^70^ and microtubule regulator STMN1.^71^ It is also noteworthy that in the colorectal cancer context, these markers are associated with increased cell proliferation and invasion. The GCLC strongly and uniquely expressed gel-forming mucin MUC2,^72^ a putative marker of the intestinal goblet cells and the building block of the mucus layer. It also highly expressed mucin MUC5AC,^73^ which is the main component of the gastric and respiratory tract mucus layer that protects the tissue from stimulations yet is absent in the intestine.^74^ Besides, part of the DEGs in adult human goblet cells were also present in the GCLC, such as IgGFc-binding protein FCGBP, SLC12A2, trefoil factor TFF3, and SPDEF (Figure 4B). Although GCLC expressed some of the most well studied functional goblet genes, part of its the DEGs were not active in adult human goblet cells nor fetal gut goblets (Figure S3B), suggesting the fundamental difference between healthy and malicious cells. Some of them, such as EGLN3,^75^ ADAM8,^76^ CA9,^77^ AQP3,^78^ and SIRT2,^79^ are related to the hypoxia response, suggesting a hypoxia-related cancer metabolism in the GCLC. Specifically, CA9 is a well-known hypoxia-inducible gene that is commonly overexpressed in cancer cells.^80^ Additional experiments, such as measuring oxygen levels in the cells would be needed to confirm if such a response is caused by the presence of hypoxia or the oncogenic signaling. Also, the intrinsic hypoxia genes expression means the Caco-2/HT29 mix might respond differently to oxygen levels or nutrient availability, so when using them as a gut epithelium mimic and co-culturing them with bacteria, their response might not be transferable to the real gut. Thus, it is essential to carefully consider the experimental conditions and controls to ensure that any observed effects are due to the bacterial co-culture and not to the Caco-2/HT29 cells' intrinsic properties. When comparing with adult human GCs, we found 1202 commonly expressed genes in GCLC were also prevalent in adult human GCs, both SI and colon (Figure 4D). Overall, the HT29-derived goblet cells were relatively comparable to its healthy adult human epithelial cells' analog. In view of the established capacity of HT29 cells to differentiate into goblet-like cells, we proposed a hypothesis concerning the developmental trajectory of U-HT29-SECPLC-GCLC. To explore this, we conducted a pseudotime analysis on select subsets (Figures 4E and 4F). The analysis revealed a notable upregulation of genes such as SOX4, AGR2, SERPINA1, and TFF3, alongside the downregulation of several others during the differentiation pathway. These findings not only elucidate the mechanisms underpinning the effective differentiation of GCLC from HT29 cells but also offer potential insights into the developmental processes associated with CRC.

Both early EECLC populations expressed immature EEC markers PAX4^21^ and INSM1^81^ (Figure 4A). EECLC-1 uniquely expressed GCG, UCN3, ETV1 (EEC subtype L-cells), SST (EEC subtype D-cells),^25^ whereas EECLC-2 expressed PROX1 (EEC subtype L-cells) and a high-level of STMN1. Most of the mature adult human EEC DEGs such as CHGA and CHGB were missing in both EECLC groups (Figure 4C), yet some EECLC DEGs were expressed in adult human EECs and more in the fetal EECs (Figure S3E). A significant number of genes (4964) present in either the adult SI or colon EECs were not universally found in EECLCs (Figure 4D), which supported our inference that EECLCs in the co-culture system were immature (i.e., had a less variated expression profile than mature EECs). Taken together, EECLCs were found in co-culture, but they might not be a perfect *in vitro* model of mature EEC.

### Co-culture model lacked TLR4, the initiator of NF-κB signaling

In the human epithelium, Lipopolysaccharide (LPS) triggers immune response like neutrophil activation by binding to Toll-like receptors (TLRs) on epithelial cells.^82^ TLR4 is primarily responsible for initiating the defense-related NF-κB signaling pathway in cells when it binds to the LPS of gram-negative bacteria.^76^ Previous studies employ Caco-2/HT29 as models to study intestinal epithelial cells' response to bacterial LPS by first confirming the presence of TLRs, or specifically TLR4. The undifferentiated HT29 is evident to express TLR4,^83^^,^^84^ but that expression in Caco-2 is controversial. Some studies find the presence of TLR4 in Caco-2 using the western blot and report more intense TLR4 expression.^84^^,^^85^ Contradictorily, other studies report little TLR4 gene expression in Caco-2, with no observed LPS response.^83^^,^^86^^,^^87^ This inconsistency is possibly due to the utilization of different anti-TLR4 and other technical differences during the cell culture. The bulk RNA-seq reads from CCLC support no TLR4 transcription in Caco-2.

In our co-culture system, neither TLR4 nor most other TLRs were detected in scRNA-seq data (Figure S2C) or using flow cytometry (Figures S1D and S1E). It is possible if 1) Caco-2, as reported, did not express TLR4, and 2) after the HT29 grew to confluence, its TLR4 expression dramatically decreased.^86^ Either way, it indicates that the co-culture model is unlikely to be stimulated by microbial products through the TLR4-NF-κB signaling pathway, which means it is a less desirable experimental model for studying the innate immune in the gut epithelium.

## Discussion

The co-culture of Caco-2/HT29 has been widely used to simulate adult human intestinal epithelium in experiments.^88^^,^^89^^,^^90^^,^^91^^,^^92^ The Caco-2 and HT29 cell lines have proven extremely useful in replicating the gut epithelium, offering researchers a valuable laboratory model for exploring gastrointestinal physiology.^6^ The Caco-2 cells, exhibiting enterocyte-like properties, primarily contribute to the understanding of absorptive processes, while the HT29 cells, characterized by mucus-secreting goblet cells, elucidate the protective mucosal barrier functions.^43^ This powerful synergistic approach has not only accelerated advancements in the study of nutrient absorption and drug delivery but has also facilitated breakthroughs in understanding host-microbe interactions when immune cell presents,^93^ the effects of cytokines and inflammatory mediators on barrier function and the expression of tight junction proteins,^94^ the adherence and invasion of pathogenic bacteria,^95^ and molecular mechanisms of colorectal cancer,^96^ including the role of genetic mutations and the effects of potential therapeutic agents.^97^

In our study, we carefully examined the variations in the Transwell co-culture, assessed how closely it resembled a healthy adult’s and fetus’ epithelium. We identified the ISC, TA, EC, GC, and EEC-like cells together with differentiating HT29 cells in the system based on the expression of canonical markers. Overall, the co-cultured cells showed less transcription variety and activities than the adult human epithelial cells, with a unique, intrinsic cancerous properties that confers the co-culture similarity toward the fetal gut given the common re-expression of embryonic genes in the colorectal adenocarcinoma. Earlier studies have compared Caco-2 and HT29 cells with the fetal colon, but due to previous limitations in transcriptomics and proteomics, only a limited number of genes were reported.^98^^,^^99^^,^^100^ Another example of discrepancy is that enterocytes-like cells in the model did not fully express the major transporters in the small intestine or colon. However, to date, none of the "major transporters" has been proven indispensable for physiological intestinal fatty acid transport.^101^ One possible explanation is that intestinal fatty acid absorption might be regulated by two or more genes so that one gene is inactivated, and the other could compensate for its function.^101^ Therefore, although the transcriptome of some transporters is absent, substances might still be able to be transported. Since in some studies the co-culture was challenged with microbes or microbial products to test its immune response, we also asked the expression of TLR4 in the co-culture and found no TLR4 expression in the co-culture. Although further experiments targeting the protein are necessary to confirm the absence of TLRs, here, we suggest that Caco-2/HT29 might not be an ideal model for studying microbe-epithelial cells interactions.

Regardless of the utility of the co-culture model, traditional animal models are often used to give a comprehensive, lifelike system for studying gut biology and confirming results from lab models. It facilitates the study of complex interactions among host genetics, immune responses, the microbiome, and different organs.^102^^,^^103^ However, the ethical considerations, costs, and potential species-specific differences limit its universal applicability. Alternative *in vitro* models, such as gut-on-a-chip, have emerged recently, presenting unique advantages and drawbacks. It offers a dynamic, microfluidic environment that incorporates fluid shear stress and mechanical strain, more accurately simulating the physiological conditions of the gut.^104^^,^^105^ Additionally, this platform allows for co-cultures of multiple cell types, including immune cells and microbes, thereby fostering a more comprehensive understanding of the intestinal ecosystem.^106^ Compared to the primary-derived experimental methods like this, Caco-2 and HT29 cells exhibit a limited gene expression profile, fetal-like characteristics, and inherent cancer properties that might deviate from the *in vivo* gut epithelium. Nevertheless, their ease of use and cost-effectiveness still make them attractive for preliminary research.

### Limitations of the study

We recognize that the primary limitation of our study stems from an under-resourced functional assay, a constraint imposed by limited funding and available resources. Despite this, by adhering to a well-established co-culturing protocol, we were able to derive a fundamental understanding of our system’s efficacy in mimicking gut physiology. This understanding is supported by two key observations: First, the formation of an intact, dense monolayer in our coculture, as visualized through brightfield microscopy, closely resembles the morphology typical of 2D *in vitro* gut models utilized in other studies. This includes both functionally characterized Caco-2/HT29 co-cultures^107^^,^^108^^,^^109^^,^^110^ and primary human intestinal monolayers derived from biopsy-based organoid cultures.^111^^,^^112^^,^^113^ Second, the transepithelial electrical resistance (TEER) values we observed, averaging between 600 and 650 Ohm cm^2^, align well with the ranges reported in previously characterized co-culture models. While we concede that additional insights could have been gleaned from employing immunofluorescence to confirm tight junction formation or Alcian blue staining for mucus layer visualization, the constraints of our setting precluded these assays. However, we partially offset these limitations through comprehensive scRNA-seq. This technique provided a robust gene expression profile, offering indirect evidence of protein expression and, by extension, functional status. The expression of cell-cell junction proteins like TJP1, OCLN, CLDN1, CLDN7, and mucin MUC2 was detected in the co-culture model, hinting at the establishment of barrier and the secretion of mucus (Figures 4A and S2C). The decision to utilize scRNA-seq was a strategic one, aimed at compensating for our inability to conduct more extensive proteomic analyses, and underscores the innovative approach of our study.

## STAR★Methods

### Key resources table

**Table:** 

REAGENT or RESOURCE	SOURCE	IDENTIFIER
**Antibodies**		

HTA125 (anti-TLR4)	BioLegend	RRID: AB_314954
MOPC-173	BioLegend	RRID: AB_326468

**Chemicals, peptides, and recombinant proteins**		

McCoy’s 5A Modified Medium	GIBCO	Cat#16600082
Dulbecco’s Modified Eagle Medium high glucose with Glutamax	GIBCO	Cat#10569069
Sodium Pyruvate	GIBCO	Cat#11360070
Penicillin-Streptomycin	GIBCO	Cat#15140122
Fetal Bovine Serum, heat inactivated	GIBCO	Cat#A5256801
Non Esential Amino Acids	GIBCO	Cat#11140050
Collagen Type I	Corning	Cat#354236
Insulin-Transferrin-Selenium	Thermo	Cat#41400045
Dimethyl Sulfoxide	Corning	Cat#25950CQC
Human TruStain FcX	BioLegend	Cat#422301
eBioscience™ Fixable Viability Dye eFluor™ 780	Thermo Fisher	Cat#65-0865-14

**Critical commercial assays**		

Single Cell 3′ Library & Gel Bead kit	10X Genomics	Cat# PN120237

**Deposited data**		

scRNA-seq (Transwell co-culture)	This paper	GEO:GSE233628
scRNA-seq (healthy adult human)	Burclaff et al.^13^	GEO:GSE185224
scRNA-seq (human fetal gut)	Chatterjee et al.^14^	https://www.gutcellatlas.org/
scRNA-seq (colorectal cancer)	Joanito et al.^15^	https://www.synapse.org/#!Synapse:syn26844071/
RNA-seq (cancer cell lines)	Cancer Cell Line Encyclopedia	https://depmap.org/portal/download/all/?release=DepMap+Public+22Q2&file=CCLE_expression_full.csv

**Experimental models: Cell lines**		

Caco-2	ATCC	HTB-37
HT-29	ATCC	HTB-38

**Software and algorithms**		

R	CRAN	4.3
Python	Python	3.8.8
cellranger	10X Genomics	6.1.2
anndata	Virshup et al., 2021	0.8.0
scanpy	Wolf et al.^114^	1.9.1
Seurat	Butler et al.^115^	4.2.0
anndata2ri	https://github.com/theislab/anndata2ri	1.1
monocle3	https://cole-trapnell-lab.github.io/monocle3/	1.3.1
Scrublet	https://github.com/swolock/scrublet	0.2.3
SCTransform	https://github.com/satijalab/sctransform	0.3.5
Matplotlib	https://matplotlib.org/	3.7.1
Seaborn	https://seaborn.pydata.org/	0.11.2
slingshot	https://bioconductor.org/packages/devel/bioc/vignettes/slingshot/inst/doc/vignette.html	2.4.0
ggplot2	Wickham, 2016	3.0.0
pheatmap	https://github.com/raivokolde/pheatmap	1.0.12
scikit-learn	https://scikit-learn.org/stable/	1.3.0
statsmodels	https://www.statsmodels.org/stable/index.html	0.14.0
numpy	https://numpy.org/	1.22.4
pandas	https://pandas.pydata.org/	2.1.0
scipy	https://scipy.org/	1.8.1
FlowJo	FlowJo, LLC	10.10

**Other**		

0.4 mm-pore polyester transwell inserts	Corning	Cat#354236
Epitehlial VOLT/OHM (TEER) meter	Worcester Polytechnic Institute	EVOM2
Attune NxT Flow Cytometer	Invitrogen	Cat#A24858

### Resource availability

#### Lead contact

Further information and requests for resources should be directed to and will be fulfilled by the lead contact, Douglas K. Brubaker (e-mail: dkb50@case.edu).

#### Materials availability

This study did not generate new unique reagents.

#### Data and code availability

• The adult human epithelial cells’ scRNA-seq data can be accessed on NCBI GEO under GSE18522414. The fetal gut epithelial cells’ scRNA-seq data can be accessed at https://www.gutcellatlas.org15, and human colorectal cancer cells’ scRNA-seq data can be accessed at https://www.synapse.org/#!Synapse:syn26844071/16. The bulk RNA-seq data of Caco-2 and HT29 can be accessed at https://depmap.org/portal/download/by searching “CCLE_expression_full.csv”. The Caco-2/HT29 coculture’s scRNA-seq data can be accessed on NCBI GEO under GSE233628.
• Script used for analysis can be found on GitHub (https://github.com/WeldonSchool-BrubakerLab/Caco2-HT29-scRNAseq).
• Any additional information required to reanalyze the data reported in this paper is available from the lead contact upon request.

### Experimental model and study participant details

#### Germ-free coculture *in-vitro* transwell gut model

The intestinal epithelial Transwell system was established by adult human colon carcinoma cell lines Caco-2 (passage no. 8) and HT29 (passage no. 13) (ATTC, United States) at 1 × 10^5^ cells/cm^2^ on permeable polyester membrane Transwell inserts. The cells were cultured in DMEM medium with Glutamax (Gibco, United States), supplemented with 10% (v/v) FBS, and incubated in a humidified atmosphere (5% CO2, 37°C) for 21 days to reach the full differentiation stage. On day 23, three Transwells were prepared for library construction following the manufacturer’s instruction. The platform used for sequencing was Illumina NovaSeq 6000 S4.

### Method details

#### Tight junction evaluation by transepithelial electrical resistance and the number of cells alive

To determine tight junction integrity, transepithelial electrical resistance (TEER) was measured by an epithelial volt-ohmmeter (EVOM2; WPI, Berlin, Germany) with STX2 Chopstick probes. Measurements were performed at each culture medium exchange according to the manufacturer’s instructions. The final TEER value was determined by subtracting the TEER measurement from a blank transwell insert and multiplying it by the cell culture surface.

#### Cell staining and flow cytometry

Caco2 and HT29 cells were harvested and stained in a cell culture treated U-bottom 96 well plate first with fixable viability dye (ef780, ThermoFisher) and human TruStain FcX (BioLegend) to block Fc receptors. The cells were then stained with anti-human TLR4 (HTA125, PE, BioLegend). Alternatively, the same cells were also stained with an isotype control antibody (MOPC-173, PE, BioLegend) or cells were left unstained (fluorescence minus one control). All samples were then washed with FACs buffer, fixed with 3.7% formaldehyde (in 1x PBS) for 10 min in the dark at room temperature, followed by washing again with FACs buffer. Cell data was then acquired with an Attune NxT Flow Cytometer (Invitrogen, Carlsbad, CA, USA). The resulting data was analyzed using the FlowJo software (v10, Eugene, OR, USA).

### Quantification and statistical analysis

#### Single cell RNA-seq data pre-processing

The FASTQ files of the sequencing data were input into the Cell Ranger^116^ pipeline by 10x genomics to be filtered and mapped to the adult human genome GRCh38 to generate a count matrix that contains the number of detected transcriptomes of each gene for each cell. The downstream analysis was done with scanpy^114^ (v1.9.1) on Python and Seurat^115^^,^^117^^,^^118^^,^^119^ (v4.2.0) on R. Each dataset was pre-processed separately. Low-quality cells were dropped based on their extremely low UMI counts, high mitochondrial gene counts, and a low number of uniquely expressed genes. In the pre-processing parameters for single-cell RNA-sequencing data, Control 1 required a minimum of 2250 unique molecular identifier (UMI) counts and at least 770 genes to be considered for analysis, while Control 2 was set with a threshold of 1500 UMI counts and a minimum of 700 genes, with both controls allowing a maximum mitochondrial gene fraction of 0.2.

Genes expressed in less than 5 cells in each dataset were excluded. Multiplets were removed by Scrublet^120^ (v0.2.3). After basic quality control, the 16019 genes' transcriptional profile in 9273 cells was obtained. The cell cycle score was calculated based on the expression of cell cycle-related genes as previously described.^121^ Each dataset was normalized by SCTransform^122^ (v0.3.5) on R. Cell’s mitochondrial fraction and the difference between the S phase score and the G2M phase score, as proposed as the representation of the cell cycle score, were regressed during the normalization. Then, the top 3000 genes that were most variable in as many datasets as possible were used as anchors to integrate the datasets in Seurat.^123^ 3000 variable features were re-calculated for the combined dataset and used to perform the principal component analysis (PCA, 50 pcs). Leiden clustering^124^ (resolution = 0.5) was performed based on the computed neighborhood graph of observations (UMAP,^125^ 50 pcs, size of neighborhood equals 15 cells) to reveal the general subtypes of the co-cultured cells. Partition-based graph abstraction^126^ (PAGA) based on the Leiden clusters was used to initialize the uniform manifold approximation (UMAP, 50 pcs, min_dist = 0.1, spread = 1.1, n_components = 2, alpha = 1.0, gamma = 1.0) to facilitate the convergence of manifold. All clusters had comparable sequencing depth and mitochondrial gene percentages (Figure S2A). Seurat integration removed the batch effect desirably (Figure S2B).

#### Subclustering and cell identity labeling

The scaled SCTransform corrected counts were used to perform the Wilcoxon rank-sum test across every Leiden cluster to find the signature of a cluster, which were genes that have log2 fold change >2 and adjusted p value <0.01. The resultant signatures, together with the epithelial markers reported previously, confirm the general identity of clusters. Each cluster was further subclustered to reveal more heterogeneity among cells. The resolution used in each subclustering is set at 0.35 for ECLC, 0.4 for Secretory, and 0.3 for U-HT29. The Wilcoxon rank-sum test was re-run on the finer clustered data to unveil the marker of each subgroup, with the same threshold for log2 fold change and adjusted p value.

#### Caco-2/HT29 similarity determination

In our study, the raw bulk mRNA sequencing (mRNA-seq) data for HT29 and Caco-2 cells were obtained from the Cancer Cell Line Encyclopedia. The sequencing reads were first rounded to the nearest integer for uniformity. To determine the differential expression between the two cell lines, we fitted the positive and negative count differences into two separate negative binomial distributions using a specialized Python script. This script utilized the fmin_l_bfgs_b function from the scipy.optimize library, optimized for maximum likelihood estimation under defined parameter bounds. This approach ensures accurate and robust fitting of the negative binomial model to our count data, considering the mean and variance of the counts for initial parameter estimation. Subsequently, we analyzed the overlap between the differentially expressed genes (DEGs) in each Leiden cluster and the Caco-2/HT29 gene signatures. This analysis was quantified using Jaccard indices, normalized against the size of the reference gene signatures. The results of this overlap analysis were visually represented through heatmaps, facilitating an intuitive understanding of the gene expression similarities and differences between the HT29 and Caco-2 cell lines.

#### CRC similarity determination

Human CRC scRNA-seq data was acquired from the previous study.^15^ It had been previously categorized into normal, iCMS2, and iCMS3 phenotypes and annotated by the original data providers. To identify the signature genes for each subtype, we performed a Wilcoxon rank-sum test across every annotated subtype using the sc.tl.rank_genes_groups function in the Python package. Signature genes were defined as those exhibiting a log2 fold change >2 and adjusted p value <0.01. Using the results, we compiled lists of upregulated genes for the iCMS2 and iCMS3 subtypes and combined these to form a comprehensive list of CRC differentially expressed genes (DEGs). We then applied SCTransform correction to scale both the co-culture counts and the human CRC counts. At this point, we employed cosine similarity, a measure used to ascertain the degree of similarity between two non-zero vectors in a multi-dimensional space, to quantify the resemblance between each co-culture and the CRC subsets. This similarity analysis, conducted using the cosine_similarity function from the sklearn.metrics.pairwise module, enabled us to effectively measure this resemblance. The resulting cosine similarity values were visualized in a heatmap, generated using the clustermap function from the seaborn library.

## Supplemental information

Document S1. Figures S1–S3

Table S1. Differentially expressed genes reported by Wilcoxon rank-sum test (log fold change >2, adjusted p < 0.05) in all clusters, related to Figure 1

Table S2. Caco-2/HT29 signatures calculated from CCLE monoculture bulk RNA-seq, related to Figure 1
