# Supplementary material for: Detailed survey of an in vitro intestinal epithelium model by single-cell transcriptomics
Source: iScience. 2024 Mar 1;27(4):109383. doi: 10.1016/j.isci.2024.109383 (PMC10959667; doi:10.1016/j.isci.2024.109383)
Supplement: Document S1. Figures S1–S3 [file mmc1.pdf]

iScience, Volume *27*

Supplemental information

Detailed survey of an *in vitro* intestinal epithelium model by single-cell transcriptomics

Ran Ran, Javier Muñoz Briones, Smrutiti Jena, Nicole L. Anderson, Matthew R. Olson, Leopold N. Green, and Douglas K. Brubaker

Fig.S1

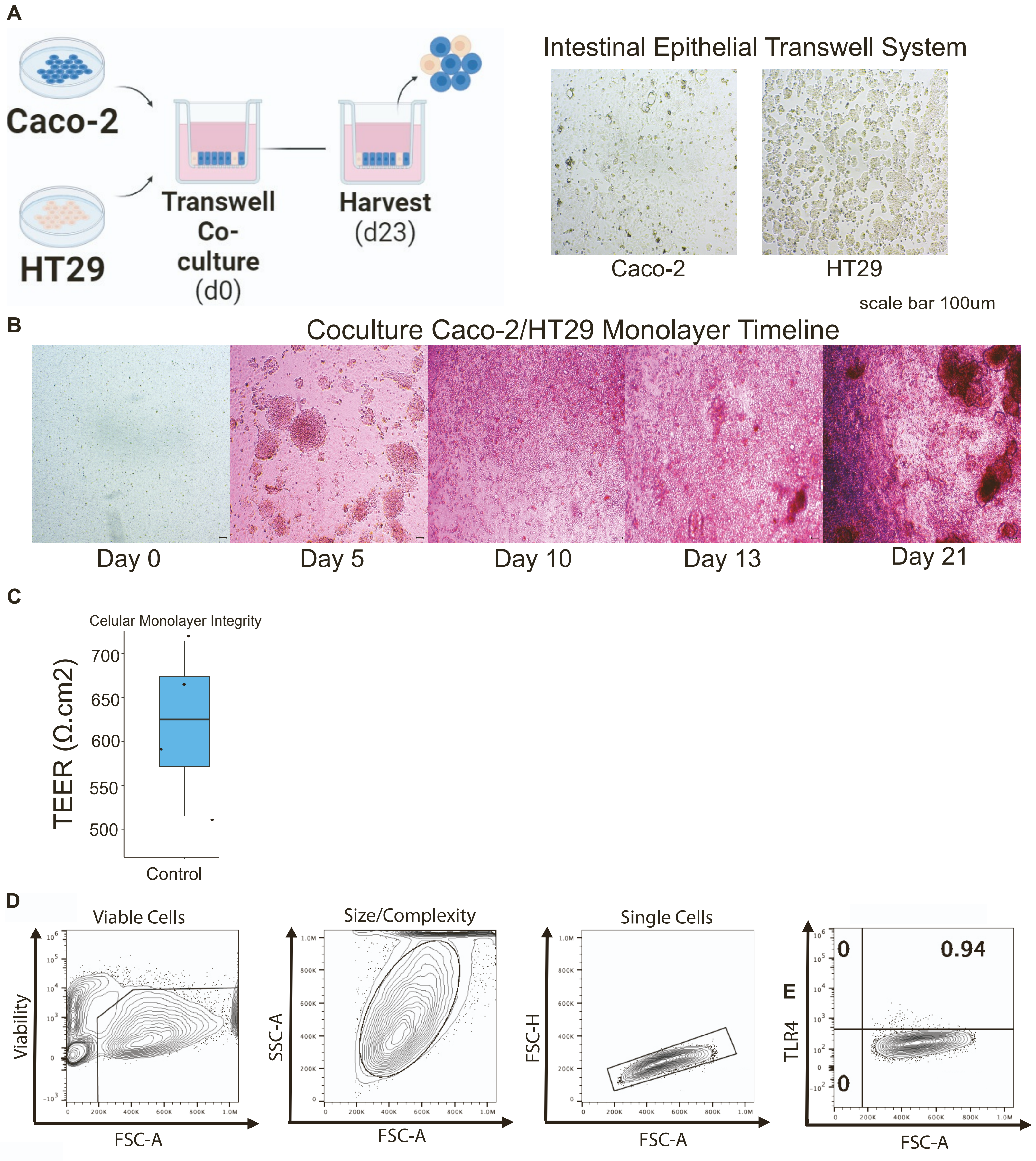

Fig. S1: Physical properties evaluation of the co-culture, related to STAR Methods.
A) Brightfield microscopy shows the Caco-2 and HT29 monoculture morphology before co-culture. B) Brightfield microscopy shows the change in morphology and confluency during the co-culture. C) Trans-epithelial/endothelial electrical resistance (TEER) measurement. D) Cell viability shown in box plot. E) TLR4 expression in HT29/Caco2 gut trans well system. Gating strategy: Viable cells are gated based on the viability dye exclusion followed by single cell selection. F) Flow cytometry contour plot showing TLR4 expression.

Fig.S2

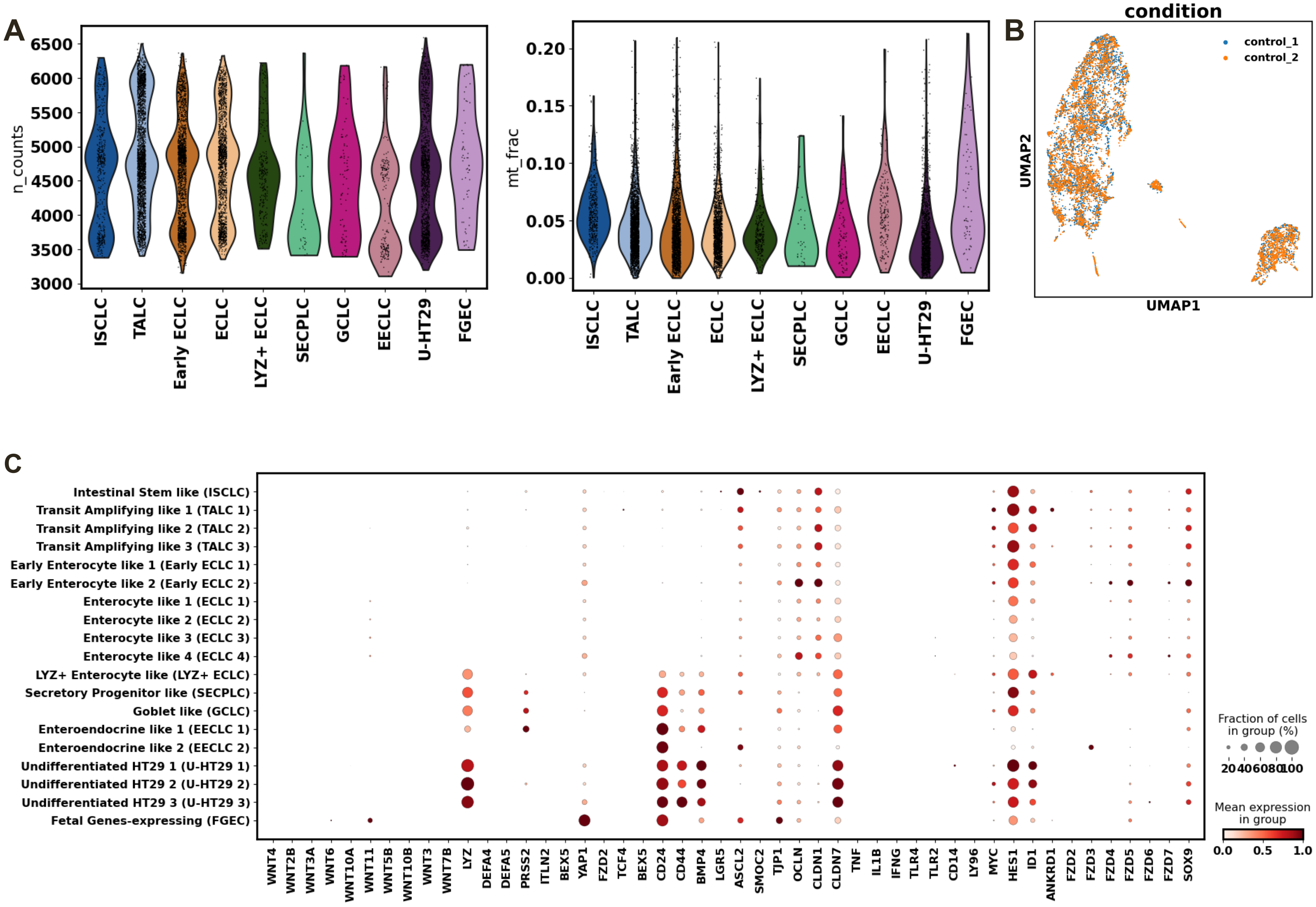

Fig. S2: Quality control and supplementary expression maps, related to Figure 1.
A) Violin plot of the sequencing depth (left, counts of mRNA per cell) and the mitochondrial fraction (right, percentage of mitochondrial mRNA in all mRNA detected in a cell) of each annotated cluster. B) UMAP showing the mixing of cells from 2 batches after batch effect removal by SCTransform. C) Dot plot showing the expression of various cell signatures as a supplement to the main figure.

Fig.S3

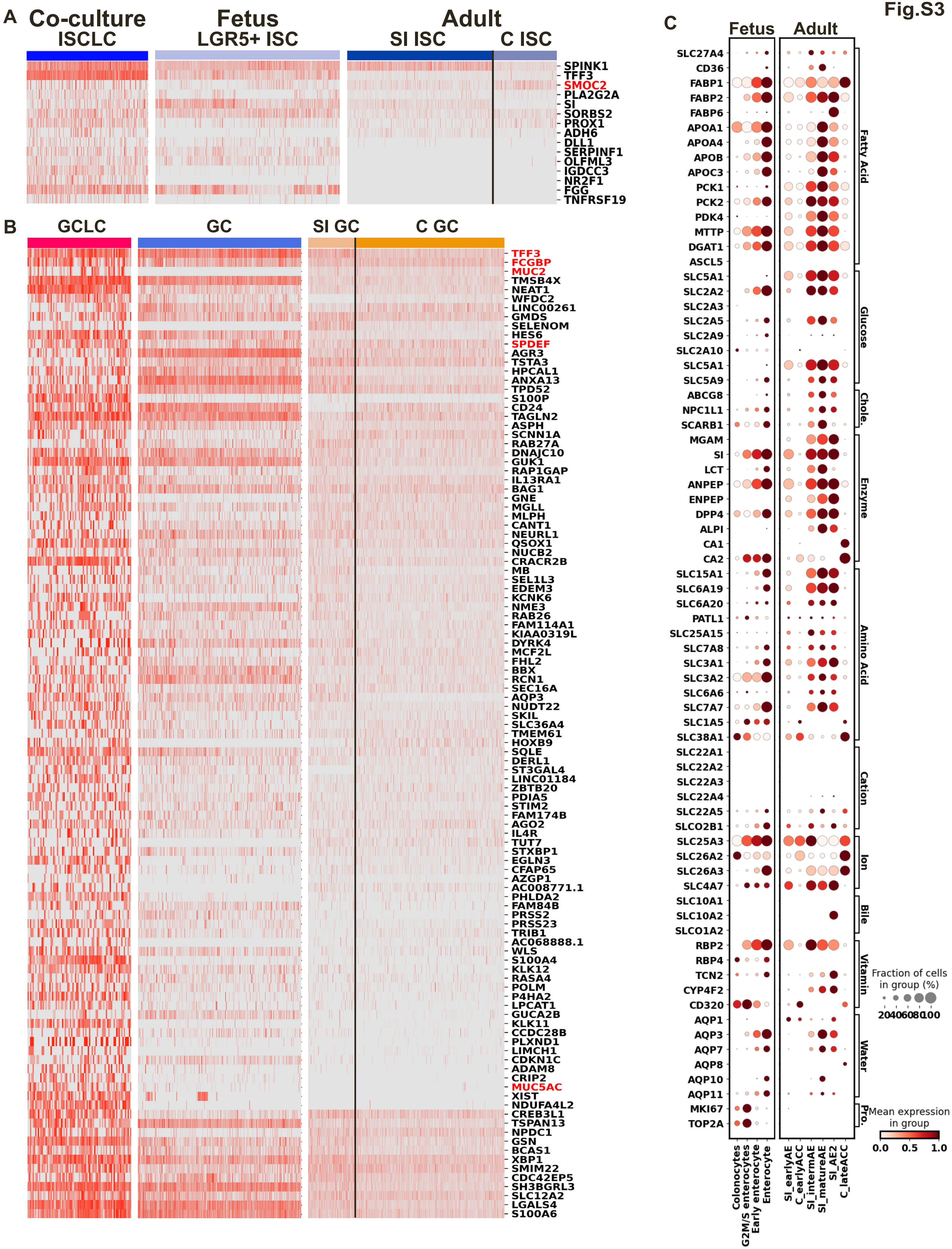
A
Co-culture
ISCLC
Fetus
LGR5+ ISC
Adult
SI ISC
C ISC
SPINK1
TFF3
SMOC2
PLA2G2A
SI
SORBS2
PROX1
ADH6
DLL1
SERPINF1
OLFML3
IGDCC3
NR2F1
FGG
TNFRSF19
B
GCLC
GC
SI GC
C GC
TFF3
FCGBP
MUC2
TMSB4X
NEAT1
WFDC2
LINC00261
GMDS
SELENOM
HES6
SPDEF
AGR3
TSTA3
HPCAL1
ANXA13
TPD52
S100P
CD24
TAGLN2
ASPH
SCNN1A
RAB27A
DNAJC10
GUK1
RAP1GAP
IL13RA1
BAG1
GNE
MGLL
MLPH
CANT1
NEURL1
QSOX1
NUCB2
CRACR2B
MB
SEL1L3
EDEM3
KCNK6
NME3
RAB26
FAM114A1
KIAA0319L
DYRK4
MCF2L
FHL2
BBX
RCN1
SEC16A
AQP3
NUDT22
SKIL
SLC36A4
TMEM61
HOXB9
SQLE
DERL1
ST3GAL4
LINC01184
ZBTB20
PDIA5
STIM2
FAM174B
AGO2
IL4R
TUT7
STXBP1
EGLN3
CFAP65
AZGP1
AC008771.1
PHLDA2
FAM84B
PRSS2
PRSS23
TRIB1
AC068888.1
WLS
S100A4
KLK12
RASA4
POLM
P4HA2
LPCAT1
GUCA2B
KLK11
CCDC28B
PLXND1
LIMCH1
CDKN1C
ADAM8
CRIP2
MUC5AC
XIST
NDUFA4L2
CREB3L1
TSPAN13
NPDC1
GSN
BCAS1
XBP1
SMIM22
CDC42EP5
SH3BGRL3
SLC12A2
LGALS4
S100A6
C
Fetus
Adult
SLC27A4
CD36
FABP1
FABP2
FABP6
APOA1
APOA4
APOB
APOC3
PCK1
PCK2
PDK4
MTTP
DGAT1
ASCL5
SLC5A1
SLC2A2
SLC2A3
SLC2A5
SLC2A9
SLC2A10
SLC5A1
SLC5A9
ABCG8
NPC1L1
SCARB1
MGAM
SI
LCT
ANPEP
ENPEP
DPP4
ALPI
CA1
CA2
SLC15A1
SLC6A19
SLC6A20
PATL1
SLC25A15
SLC7A8
SLC3A1
SLC3A2
SLC6A6
SLC7A7
SLC1A5
SLC38A1
SLC22A1
SLC22A2
SLC22A3
SLC22A4
SLC22A5
SLCO2B1
SLC25A3
SLC26A2
SLC26A3
SLC4A7
SLC10A1
SLC10A2
SLCO1A2
RBP2
RBP4
TCN2
CYP4F2
CD320
AQP1
AQP3
AQP7
AQP8
AQP10
AQP11
MKI67
TOP2A
Fatty Acid
Glucose
Chole.
Enzyme
Amino Acid
Cation
Ion
Bile
Vitamin
Water
Pro.
Colonocytes
G2M/S enterocytes
Early enterocyte
Enterocyte
SI_earlyAE
C_earlyACC
SI_intermAE
SI_matureAE
SI_AE2
C_lateACC
Fraction of cells
in group (%)
20406080100
Mean expression
in group
0.0
0.5
1.0

Fig.S3

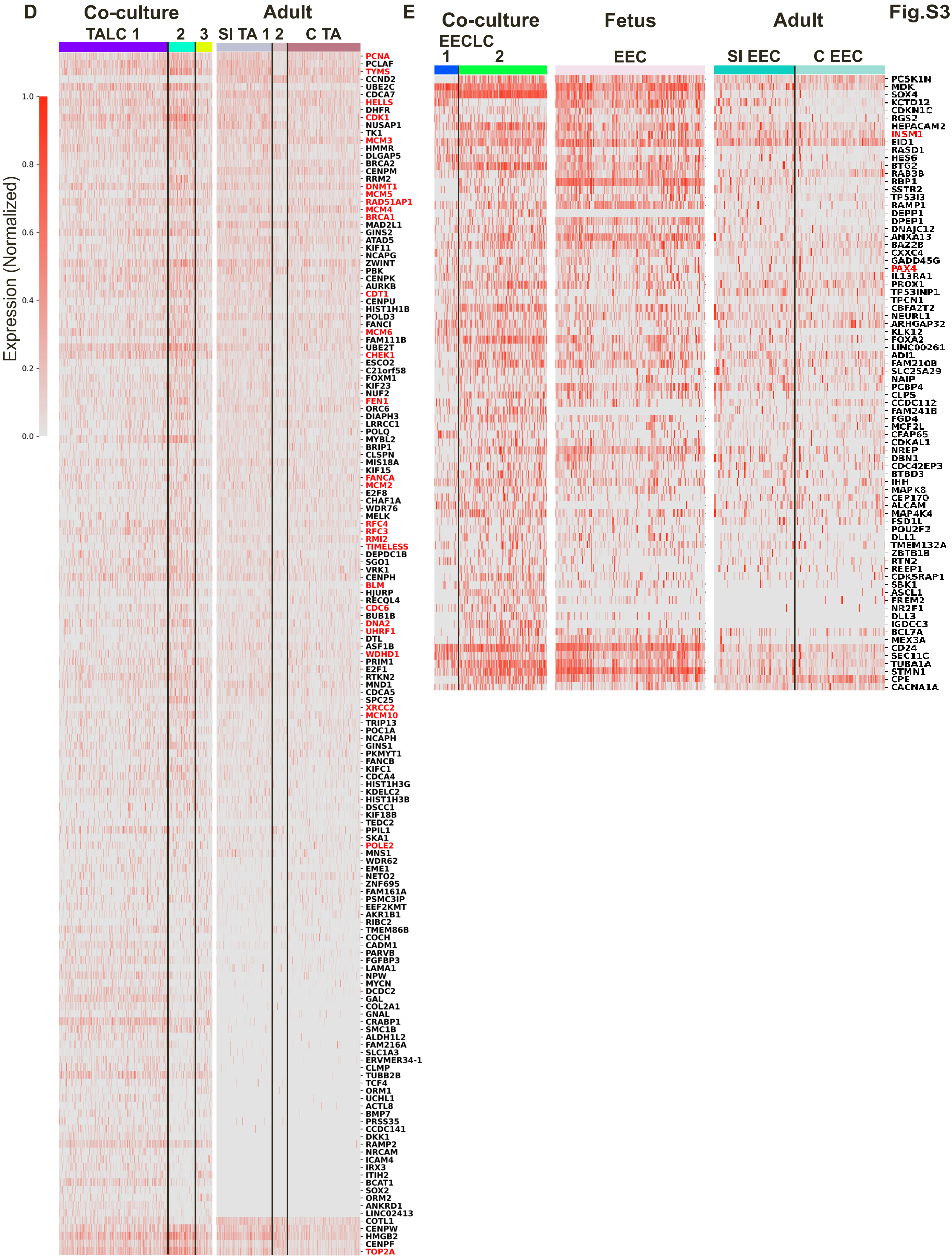

D
Co-culture
TALC 1
2
3
Adult
SI TA 1
2
C TA
Expression (Normalized)
1.0
0.8
0.6
0.4
0.2
0.0
PCNA
PCLAF
TYMS
CCND2
UBE2C
CDCA7
HELLS
DHFR
CDK1
NUSAP1
TK1
MCM3
HMMR
DLGAP5
BRCA2
CENPM
RRM2
DNMT1
MCM5
RAD51AP1
MCM4
BRCA1
MAD2L1
GINS2
ATAD5
KIF11
NCAPG
ZWINT
PBK
CENPK
AURKB
CDT1
CENPU
HIST1H1B
POLD3
FANCI
MCM6
FAM111B
UBE2T
CHEK1
ESCO2
C21orf58
FOXM1
KIF23
NUF2
FEN1
ORC6
DIAPH3
LRRCC1
POLQ
MYBL2
BRIP1
CLSPN
MIS18A
KIF15
FANCA
MCM2
E2F8
CHAF1A
WDR76
MELK
RFC4
RFC3
RMI2
TIMELESS
DEPDC1B
SGO1
VRK1
CENPH
BLM
HJURP
RECQL4
CDC6
BUB1B
DNA2
UHRF1
DTL
ASF1B
WDHD1
PRIM1
E2F1
RTKN2
MND1
CDCA5
SPC25
XRCC2
MCM10
TRIP13
POC1A
NCAPH
GINS1
PKMYT1
FANCB
KIFC1
CDCA4
HIST1H3G
KDELC2
HIST1H3B
DSCC1
KIF18B
TEDC2
PPIL1
SKA1
POLE2
MNS1
WDR62
EME1
NETO2
ZNF695
FAM161A
PSMC3IP
EEF2KMT
AKR1B1
RIBC2
TMEM86B
COCH
CADM1
PARVB
FGFBP3
LAMA1
NPW
MYCN
DCDC2
GAL
COL2A1
GNAL
CRABP1
SMC1B
ALDH1L2
FAM216A
SLC1A3
ERVMER34-1
CLMP
TUBB2B
TCF4
ORM1
UCHL1
ACTL8
BMP7
PRSS35
CCDC141
DKK1
RAMP2
NRCAM
ICAM4
IRX3
ITIH2
BCAT1
SOX2
ORM2
ANKRD1
LINC02413
COTL1
CENPW
HMGB2
CENPF
TOP2A
E
Co-culture
EECLC
1
2
Fetus
EEC
Adult
SI EEC
C EEC
PCSK1N
MDK
SOX4
KCTD12
CDKN1C
RGS2
HEPACAM2
INSM1
EID1
RASD1
HES6
BTG2
RAB3B
RBP1
SSTR2
TP53I3
RAMP1
DEPP1
DPEP1
DNAJC12
ANXA13
BAZ2B
CXXC4
GADD45G
PAX4
IL13RA1
PROX1
TP53INP1
TPCN1
CBFA2T2
NEURL1
ARHGAP32
KLK12
FOXA2
LINC00261
ADI1
FAM210B
SLC25A29
NAIP
PCBP4
CLPS
CCDC112
FAM241B
FGD4
MCF2L
CFAP65
CDKAL1
NREP
DBN1
CDC42EP3
BTBD3
IHH
MAPK8
CEP170
ALCAM
MAP4K4
FSD1L
POU2F2
DLL1
TMEM132A
ZBTB18
RTN2
REEP1
CDK5RAP1
SBK1
ASCL1
FREM2
NR2F1
DLL3
IGDCC3
BCL7A
MEX3A
CD24
SEC11C
TUBA1A
STMN1
CPE
CACNA1A

Fig. S3: Heatmaps of co-culture differentially expressed genes expression in co-culture, fetus, and adult, along with the common nutrients transporters and enzymes expression in fetus and healthy adult showing in a dot plot, related to Figure 2, 3, 4.

A) Intestinal stem cells (ISC) in fetus and adult compared to ISC-like cells (ISCLC) in co-culture. SI: small intestine. C: colon. B) Goblet cells (GC) in fetus and adult compared to GC-like cells (GCLC) in co-culture. C) The expression of various nutrients transporters and enzymes in fetal and adult enterocytes/colonocytes as a reference of Fig. 3A. AE: absorptive enterocytes. ACC: absorptive colonocytes. D) Transit amplifying (TA) cells in adult compared to TA-like cells (TALC) in co-culture. E) Enteroendocrine cells (EEC) in fetus and adult compared to EEC-like cells (EECLC) in co-culture.
